# Water-Use Efficiency for Post-Weaning Growth Performance of South African Beef Cattle Under Intensive Production Systems

**DOI:** 10.3390/ani15172505

**Published:** 2025-08-26

**Authors:** Ayanda M. Ngxumeshe, Takalani Mpofu, Khathutshelo Nephawe, Motshekwe Ratsaka, Bohani Mtileni

**Affiliations:** Department of Animal Sciences, Tshwane University of Technology, Pretoria 0001, South Africa; mpofutj@tut.ac.za (T.M.); nephaweka@tut.ac.za (K.N.); ratsakamm@tut.ac.za (M.R.); mtilenib@tut.ac.za (B.M.)

**Keywords:** sustainable production, resource use, environmental impact

## Abstract

This study determined the water-use efficiency for post-weaning growth performance of beef cattle of different frame sizes under intensive production systems. A total of 33 beef cattle weaners of three different frame sizes (small, medium, and large) were randomly allocated individually to metabolic pens. Feed and water were provided ad libitum. The water intake (WI), feed intake (FI), and weight were measured across different feeding phases (starter, grower, and finisher). Water consumption (WC) average daily gain (ADG), weight gain (WG), water intake efficiency (WIE), water footprint per animal (WFP/AU), and WFP/kg were computed. General Linear Model of Statical Analysis software (SAS) version 9.4 was used to analyse the data, and the means were separated using Fisher’s LSD test. The results showed that large-frame beef cattle had significantly higher (*p* < 0.05) WT_f_. (412.73 ± 27.27 kg) and WI (3394.09 ± 156.3 L), but also the largest WFP/AU (4407 ± 197.22 L). The medium-frame cattle achieved the highest ADG (1.48 ± 0.14 kg/day) and a moderate WIE (20.15 ± 2.18 L/kg gain), indicating an optimal trade-off between productivity and water use. The small-frame beef cattle exhibited the best WCE (0.051 ± 0.005 kg/L) and the lowest WFP/AU (3822 ± 197.22 L), highlighting superior water-use adaptability. Pearson’s correlation revealed that WCE was positively associated with ADG (r = 0.499; *p* < 0.05) and negatively with WIE (r = −0.987; *p* < 0.05). These findings suggest that medium-frame beef cattle provided a balanced compromise between growth performance and resource efficiency, making them more suitable for sustainable production in water-limited environments.

## Simple Summary

Water scarcity poses a serious challenge to sustainable beef production in South Africa, especially under intensive feeding systems. This study evaluated water-use efficiency among three beef cattle frame sizes, including small (Nguni), medium (Bonsmara), and large (Simmental), during the post-weaning feeding phase. Thirty-three weaners were individually fed and monitored for feed intake, water intake, weight gain, and various water efficiency indicators, including water intake efficiency, water consumption efficiency, and water footprint per animal unit. The results showed that the large-frame beef cattle achieved the highest weight gains but consumed the most water, resulting in the largest water footprint. Small-frame beef cattle used water more efficiently, while medium-frame beef cattle demonstrated the best balance between growth performance and water-use efficiency. Correlation analyses confirmed strong relationships between water-use indicators and growth traits. These findings support the selection of medium-frame beef cattle for optimizing both productivity and water efficiency in resource-limited environments. This research contributes to improving the environmental sustainability of intensive beef systems, aligning with global efforts to meet the Sustainable Development Goals related to food security, water conservation, and climate change adaptation.

## 1. Introduction

The beef industry in South Africa has come under scrutiny due to its high water footprint (WF), which comprises both direct and indirect water inputs used in feed production, drinking, and service operations [1,2]. In intensive beef production systems, optimizing water use without compromising growth performance has become an urgent priority, especially in the face of escalating water scarcity, climate variability, and competing water demands across sectors [3,4]. Post-weaning growth is a critical phase in beef cattle development, marked by high nutritional and water demands to support skeletal growth, muscle accretion, and metabolic activity [5,6]. During this period, water intake (WI) is directly linked with feed intake (FI) and nutrient metabolism, which directly affect body weight gain (WG) and overall performance. Yet, few studies have systematically evaluated water-use efficiency (WUE) indicators in the context of post-weaning growth, particularly under intensive feeding conditions that aim to maximize productivity per unit of input.

Water-use efficiency indicators help differentiate animals with superior adaptive capacities and can inform selection programs targeting both performance and resource-use sustainability [7,8]. Research has shown that variations in WI and utilization efficiency exist among different beef cattle frame sizes and production environments [3,9,10], necessitating a deeper evaluation of WUE at various feeding phases [8,11]. Ahlberg et al. [10] and Pereira et al. [12] reported that WI is positively associated with FI, which is typically higher in large-frame beef cattle due to their greater nutrient requirements and longer growth cycles. Small-frame beef cattle were reported to have higher efficiency of resource utilization, including water and feed, translating to reduced overall water footprint due to shorter feedlot durations and lower cumulative inputs [13,14]. While Ridoutt et al. [15] and Terry et al. [16] emphasized that medium-frame beef cattle can optimize productivity without disproportionately increasing water demands, Palhares et al. [8] found that animal-level management, including choosing an optimal frame size, significantly reduces the water footprint of beef production systems.

However, these metrics remain underutilized in South African beef production research, where genotype–environment interactions and production intensification are creating complex demands on animal performance and resource management [17]. The current study aimed to determine the water-use efficiency indicators and their relationship with post-weaning growth performance in South African beef cattle under intensive production systems. While this research supports the goals of Sustainable Development Goal 6 (clean water and sanitation) and SDG 12 (responsible consumption and production), the primary motivation lies in generating locally relevant, data-driven insights to guide sustainable livestock management. By identifying performance-linked WUE patterns across different beef cattle frame sizes, this study contributes to the development of precision livestock management strategies that enhance productivity while reducing environmental impact.

## 2. Materials and Methods

### 2.1. Experimental Design and Animal Feeds

This study was conducted at the animal nutrition section of the Agricultural Research Council—Production Institute (ARC-PI) in Pretoria, Gauteng, South Africa (25°53′59.6″ S and 28°12′51.6″ E). The area is characterized by an ambient temperature range of 18 to 29 °C during summer and between 5 and 20 °C during winter. Thirty-three (33) beef cattle weaners of three different body frame sizes (large, medium and small), representing three different breeds (Simmental, Bonsmara, and Nguni, respectively) of similar age and body weight groups (150–265 kg), were obtained from stud breeders. On arrival at the farm, animals were ear-tagged and vaccinated. The animals were randomly assigned to treatments in a completely randomised design, i.e., eleven (11) animals per body frame size, and each animal was a replicate unit. The animals were allowed a 28-day adaptation period, followed by data collection for 90 days, over three feeding phases (starter (30 days), grower (32 days) and finisher (28 days)), with the animals placed individually in feeding pens. The diets were formulated based on hominy chop (a maize by-product) and soybean oilcake, providing a high-energy, moderate-protein ration suitable for post-weaning growth (Table 1). The formulation reflects a typical maize–soy-based intensive feedlot diet, balanced for energy (TDN > 74%), protein (CP~13.5%), and essential minerals, with roughage provided by Eragrostis hay. Each formulation was calculated per 1000 kg of total mixed ration on an as-fed basis.

### 2.2. Performance Measurements

At the beginning and end of the trial, then at two-week intervals during the trial, the animals were weighed using a platform electronic cattle-weighing scale (Ruddweigh RW1500, Ruddweigh International Pty Ltd., Guyra, Australia). This interval was selected as it allows for the detection of noticeable differences in body weight associated with diet changes, without unnecessary animal handling stress. Weight gain was determined by subtracting the weight at the beginning of each growth period from the weight at the end of the growth period. The average daily gain (ADG) was determined by subtracting the initial body weight from the final body weight and dividing by the number of days of the feeding phase. Feed was offered ad libitum weekly, and refusals were collected and weighed before fresh feed was provided. Since each animal was housed in an individual pen, FI and refusals were measured on an individual basis. The feed conversion ratio (FCR, kg FI/kg WG) was estimated by dividing FI by WG. The following equations were used for all the performance measures computed:

(1) $$FI=F_{in}-F_{out}$$

(2) $$WG={Wt}_{f}\;-{Wt}_{i}$$

(3) $$ADG=\frac{WG\;(kg)}{\#\;Days}$$

(4) $$FCR=\frac{FI\;(kg)}{WG\;(kg)}$$

where Wt_i_—initial weight at the beginning of the experimental period and each feeding period.

Wt_f_—final weight at the end of each feeding period.
F_in_—feed weighed in at the beginning of the week.
F_out_—feed weighed out of feeders at the end of the week.

### 2.3. Water Efficiency Measures

Water was provided ad libitum to all animals using individual calibrated water troughs with water meter readings. Water intake (WI) of individual animals was measured daily at 08H00 in the morning before feeding. Water consumption was calculated by adding water intake to the water in the feed. The moisture content of the feed was determined using the oven-drying method according to AOAC [18], Method 934.01. Water intake efficiency (WIE) was calculated as the ratio of WI to the weight gain (WG) of the animal. Water consumption efficiency (WCE) was calculated as the ratio of live weight gain of the animal to the total volume of water consumed (kg weight gained per litres of water consumed) [10,12]. The water footprint of a live animal consists of different components: the indirect water footprint of the feed and the direct water footprint of both drinking and service water [1,19] were computed. Service water included water used for cleaning animal drinking troughs. The volume of service water used was recorded weekly using a calibrated water meter and was evenly allocated across animals based on animal unit (AU). The following water-use efficiencies were computed:

(5) $$WFR=\frac{WI\;(L)}{FI\;(kg)}$$

(6) $$WIE=\frac{WI\;(L)}{ADG\;(kg)}$$

(7) $$WCE=\frac{ADG\;(kg)}{WC\;(L)}$$

(8) $${WFP}_{blue}/kg=\frac{{WC+W}_{serv.}}{WG}$$

(9) $${WFP}_{blue}={WC+W}_{serv.}$$

where WFP_blue_—the blue water footprint.

W_serv._—service water used for cleaning drinking troughs.

### 2.4. Statistical Analysis

The water-use efficiency data were analysed using repeated measures techniques of SAS 9.4 [20] in PROC MIXED considering the covariance structure of the observed data. The following statistical model was used:

(10) $$Y_{ijk}=\mu +T_{i}+W_{k}+{\left(TW\right)}_{ik}+\varepsilon_{ijk}$$

where $Y_{ijk}$ = measurement of response (Wt_i_, Wt_f_, WG, ADG, FI, FCR, WI, WC, WIE, WCE, WFR, WFP/AU and WFP/kg when the time was included as a classification variable) on the *j*th herd of the ith frame size treatment (small, medium, and large) at the *k*th time (feeding phase); μ = overall mean; T_i_ = fixed effect of beef cattle frame size (small, medium and large); W_k_ = fixed effect of the *k*th time on measurements (*k* = 1, 2, 3); (TW)_ik_ = interaction between *i*th frame sizes and *k*th time; $\varepsilon_{ijk}$ = random error associated with the *k*th animal in the *i*th frame size at the *j*th time.

Several covariance structures—including compound symmetry (CS), autoregressive [AR(1)], and unstructured (UN)—were evaluated to model the correlation of repeated measures within individual animals. Model selection was guided by comparisons of the Akaike Information Criterion (AIC) and Bayesian Information Criterion (BIC) values. The AR(1) covariance structure provided the best fit (i.e., lowest AIC and BIC) and was therefore used in the final model.

The mean separation was conducted using Fisher’s LSD test (*p* < 0.05). Pearson’s product–moment correlation coefficient (*r*) was computed to determine the relationship between water footprint indicators and growth performance traits, as follows:

(11) $$r=\frac{\sum (X_{i}-\bar{X})(Y_{i}-\bar{Y})}{\sqrt{\sum {\left(X_{i}-\bar{X}\right)}^{2}\sum {\left(Y_{i}-\bar{Y}\right)}^{2}}}$$

The significance of correlation was tested using the *t*-test for Pearson’s correlation:

(12) $$r=\frac{r\sqrt{n-2}}{\sqrt{1-r^{2}}}$$

where n is the sample size. A *p*-value < 0.05 was considered statistically significant.

## 3. Results

### 3.1. Growth Performance, Feed and Water Intake

Table 2 presents the means and least significant differences (LSDs) of growth performance of beef cattle under intensive production systems. The initial weight (Wt_i_.) was significantly (*p* < 0.05) lower in small-frame beef cattle (159.77 kg) compared to medium-frame (228.41 kg) and large-frame beef cattle (265.14 kg). The large-frame beef cattle reached significantly (*p* < 0.05) higher WT_f_ (412.73 kg), followed by the medium-frame (383.46 kg) and small-frame beef cattle. The large-frame and medium-frame beef cattle consumed significantly (*p* < 0.05) more feed (1021.59 kg and 1025.21 kg, respectively), compared to the small-frame beef cattle (813.68 kg). The large-frame beef cattle had significantly (*p* < 0.05) higher WI (3394.09 L) and WC (3471.88 L), followed by the medium-frame (WI: 3095.64 L and WC: 3174.07 L) and small-frame beef cattle (WI: 2510.64 L and WC: 2572.88 L). Weight gain was significantly (*p* < 0.05) lower for the small-frame (132.36 kg) compared to the medium-frame beef cattle (155.05 kg). For the large-frame beef cattle, WG was not significantly (*p* > 0.05) different from both the small-frame and medium-frame beef cattle. The medium-frame beef cattle had a higher ADG (1.48 kg/day), significantly (*p* < 0.05) outperforming the small-frame (1.26 kg/day). Feed conversion ratio (FCR) and WFR did not differ significantly (*p* > 0.05) among beef cattle frame sizes. The large-frame beef cattle exhibited significantly (*p* < 0.05) higher WIE (23.15 L/kg) compared to the small-frame (19.37 L/kg) and medium-frame (20.15 L/kg) beef cattle. The small-frame and medium-frame beef cattle showed significantly (*p* < 0.05) higher WCE (0.051 kg/L and 0.049 kg/L, respectively) compared to the large-frame beef cattle (0.042 kg/L). No significant (*p* > 0.05) differences were observed in WFP/kg. The large-frame beef cattle had significantly (*p* < 0.05) higher WFP/AU (4407 L) than the medium-frame (4185 L) and small-frame (3822 L) beef cattle.

**Figure 1:**
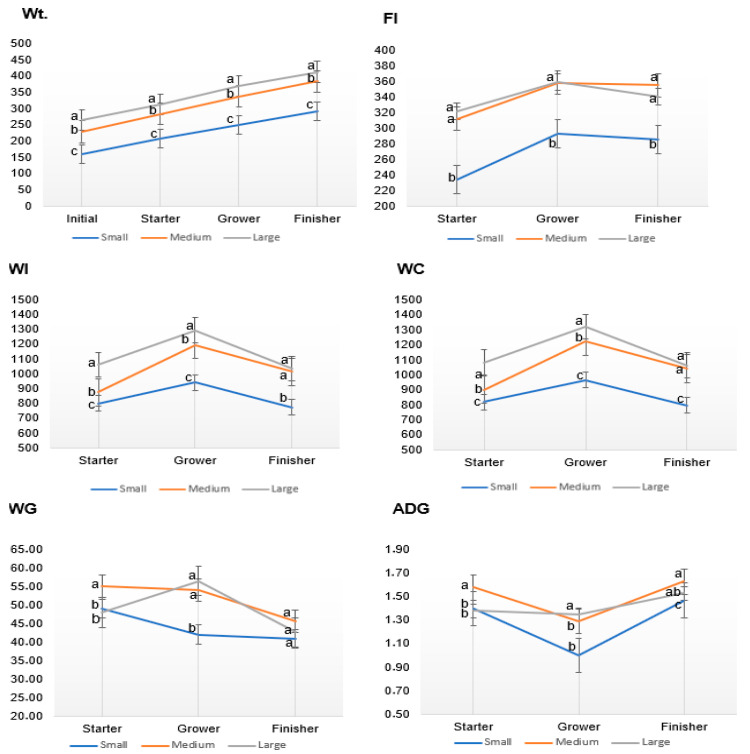
Performance traits (Wt: final weight, FI: feed intake, WI: water intake, WC: water consumption, WG: weight gain, ADG: average daily gain) of beef cattle under intensive production systems over different feeding phases (starter, grower, and finisher). Different superscripts indicate significant difference at *p* < 0.05.

Figure 1 presents the growth performance of three beef cattle frame sizes across feeding phases. The large-frame beef cattle consistently showed significantly (*p* < 0.05) higher weights at each phase (starter: 313.32 kg; grower: 370 kg; and finisher: 412.73 kg), followed by the medium-frame beef cattle (starter: 283.59 kg; grower: 337.73 kg; and finisher: 383.45 kg) and the small-frame beef cattle (starter: 251.09 kg; grower: 292.14 kg; and finisher: 283.59 kg). Weight gain (WG) was significantly (*p* < 0.05) higher during the starter and grower periods for all frame sizes, and the finisher period showed lower WG. The medium-frame beef cattle showed the highest ADG across all periods. The ADG for the small-frame beef cattle was significantly (*p* < 0.05) lower during the grower phase (1.003 kg). Feed intake increased during the grower period and declined during the finisher period across all frame sizes. The medium-frame and large-frame beef cattle had higher FI (starter: 311.86 kg; grower: 358.32 kg; and finisher: 355.03 kg) and (starter: 321.92 kg; grower: 358.95 kg; and finisher: 340.72 kg), respectively, compared to the small-frame beef cattle (starter: 234.39 kg; grower: 293.29 kg; and finisher: 285.70 kg). Both WI and WC were higher during the grower period for all beef cattle frame sizes. The large-frame beef cattle consumed significantly (*p* < 0.05) more water than the small-frame and medium-frame beef cattle across all periods.

**Figure 2:**
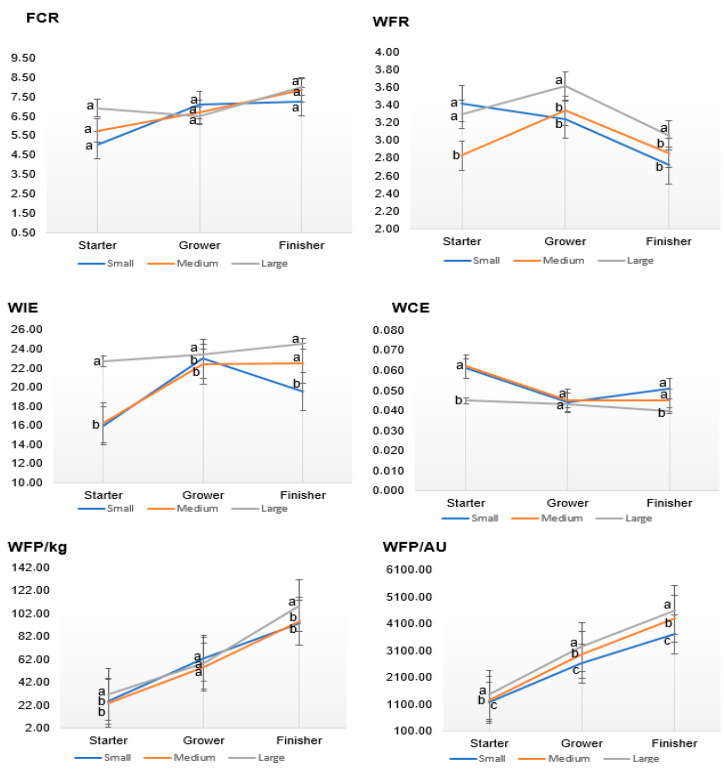
Efficiency measures of beef cattle over time (FCR: feed conversion ratio, WFR: water-to-feed ratio, WIE: water intake efficiency, WCE: water consumption efficiency, WFP/kg: water footprint per kilogram gain, WFP/AU: water footprint per animal unit). Different superscripts indicate significant difference at *p* < 0.05.

Figure 2 presents the efficiency measures of beef cattle across growth periods. The small-frame beef cattle had a significantly (*p* < 0.05) lower FCR (5.03) during the starter period, followed by the medium-frame (5.75) and large-frame (6.93) beef cattle. No significant differences (*p* > 0.05) observed with FCR for all frame sizes during the grower and finisher growth phase. The large-frame beef cattle had significantly (*p* < 0.05) higher WFR across all growth phases. During the starter growth phase, the small (3.42) and large (3.30) frame size beef cattle had significantly (*p* < 0.05) higher WFR than the medium-frame beef cattle (2.83). There were no significant differences (*p* > 0.05) noted during the grower growth phase. The large-frame beef cattle had a significantly (*p* < 0.05) higher WFR (3.06) than the medium (2.86) and small (2.72) frame size beef cattle.

The large-frame beef cattle had a significantly (*p* < 0.05) lower WIE during the starter growth phase (22.73 L/kg) compared to the medium (16.56 L/kg) and small-frame beef cattle (16.99 L/kg). Small and medium-frame beef cattle had significantly (*p* < 0.05) higher WCE than the large-frame beef cattle (0.045 kg/L) during the starter (0.061 kg/L and 0.062 kg/L, respectively) and finisher (0.051 kg/L and 0.040 kg/L, respectively) growth phases compared the large-frame beef cattle (0.045 kg/L and 0.040 kg/L for starter and finisher phases respectively). During the grower growth phase, WCE had no significant (*p* > 0.05) among the beef cattle frame sizes. Large-frame beef cattle had significantly (*p* < 0.05) higher WFP/AU across all growth phases. During the finisher phase, the large-frame beef cattle had higher WFP/AU (4602 L), followed by the medium (4295 L) and the small-frame beef cattle (3712 L). The WFP/kg increased significantly over the growth phases. During the starter feeding phase, the small (25.36 L/kg) and medium (23.58 L/kg) had significantly (*p* < 0.05) lower WFP/kg than the large-frame beef cattle (31.27 L/kg). During the grower growth phase, WFP/kg was not significantly different (*p* > 0.05) across all beef cattle frame sizes. The large-frame beef cattle had significantly (*p* < 0.05) high WFP/kg (108.77 L/kg) during the finisher phase compared to the medium and small beef cattle frame sizes.

### 3.2. Correlations

**Figure 3:**
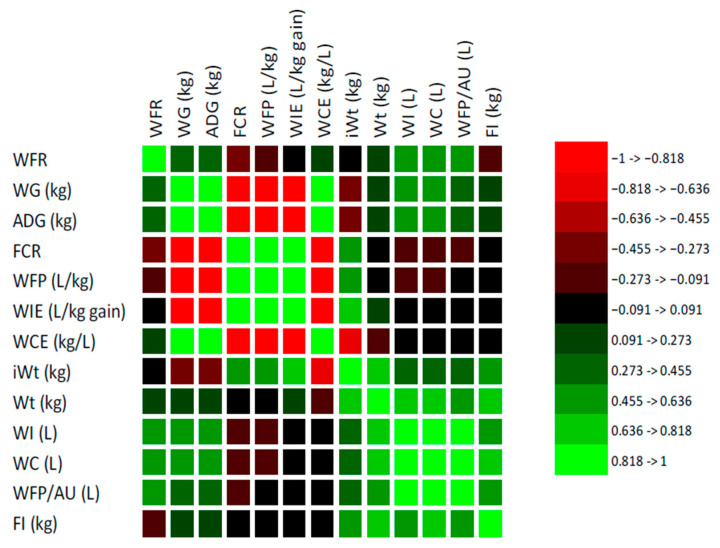
Pearson correlation analysis between water-use indicators and performance traits of the combined beef cattle frame sizes during the post-weaning feeding period under intensive production.

The Pearson correlation analysis revealed significant interrelationships between water-use indicators and performance traits of the combined beef cattle frame sizes during the post-weaning growth phase under intensive production (Figure 3). Water consumption efficiency (WCE) exhibited a moderate positive correlation with ADG (r = 0.499; *p* < 0.05) and WG (r = 0.499; *p* < 0.05), indicating that animals with superior growth performance utilized water more efficiently. In contrast, WCE was significantly (*p* < 0.05) and negatively correlated with WIE (r = −0.987), FCR (r = −0.886), and WFP/kg (r = −0.828), implying that improved efficiency reduces overall water demand and environmental impact per unit of meat produced. Water intake efficiency (WIE) and FCR were positively and strongly correlated (r = 0.890; *p* < 0.05), and both variables correlated with WFP/kg (r = 0.847 and 0.844, respectively). Both WIE and FCR were negatively correlated with ADG (r = −0.520) and WG (r = −0.594), emphasizing that fast-growing cattle tend to be more resource efficient. Water footprint per kilogram (WFP/kg) was significantly and negatively correlated with ADG (r = −0.835; *p* < 0.05) and WG (r = −0.835; *p* < 0.05), confirming that animals that gain weight more rapidly reduce the amount of water needed per kilogram of beef produced. Interestingly, WFP/AU correlated positively with WI (r = 0.923), WC (r = 0.923), and final body weight (r = 0.869), all statistically significant (*p* < 0.05). Feed intake (FI) correlated strongly with WI (r = 0.884, *p* < 0.05), WC (r = 0.889, *p* < 0.05), and final Wt. (r = 0.931, *p* < 0.05), but showed only weak correlations with FCR (r = 0.275, *p* > 0.05) and WIE (r = 0.276, *p* > 0.05). The WFR showed a moderate positive correlation with WIE (r = 0.517, *p* < 0.05) and WI (r = 0.514, *p* < 0.05) and a negative correlation with WCE (r = −0.507, *p* < 0.05).

**Figure 4:**
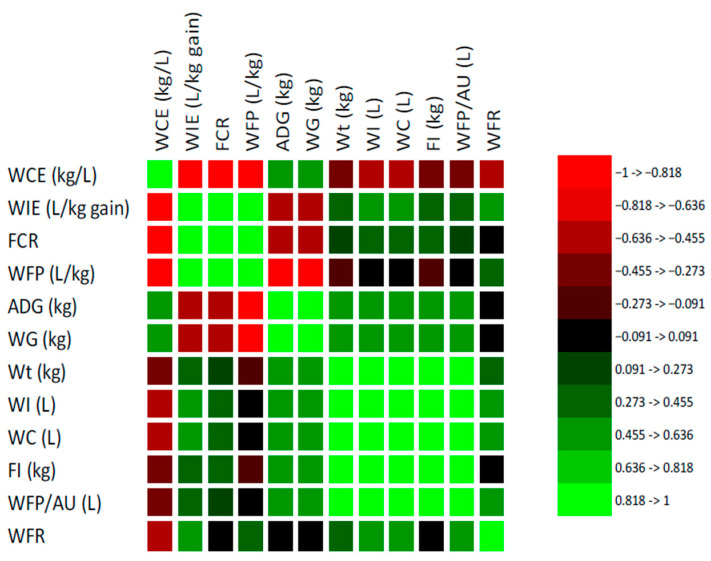
Pearson correlations between water metrics and performance traits in small-frame beef cattle under intensive production systems.

The Pearson correlation analysis revealed several significant correlations between water and performance parameters for small-frame beef cattle under intensive production systems (Figure 4). A strong and positive correlation was observed between WI and WC (r = 1.000; *p* < 0.05). Similarly, WI showed strong positive correlations with WFP/AU (r = 0.911; *p* < 0.05) and FI (r = 0.634; *p* < 0.05). Water consumption efficiency (WCE) was strongly and positively correlated with WG (r = 0.890; *p* < 0.05) and ADG (r = 0.890; *p* < 0.05). Conversely, strong negative correlations were found between feed conversion ratio (FCR) and both WG and ADG (r = −0.931; *p* < 0.05), indicating that as feed efficiency improves (i.e., lower FCR), weight gains increase. In contrast, FCR was positively correlated with water footprint (WFP; r = 0.935) and WIE (r = 0.930; *p* < 0.05). Both WFP and WIE were strongly and negatively associated with WCE (r = −0.986 and −0.984, respectively; *p* < 0.05), affirming that higher water input per unit of gain leads to diminished water-use efficiency. Moreover, WFP and WIE exhibited strong positive correlations with FCR, reinforcing the interdependence between feed and water conversion metrices. Final Wt. was positively correlated with total WI (r = 0.776; *p* < 0.05). Furthermore, final Wt. was positively correlated with FI (r = 0.788; *p* < 0.05). Finally, WFP/AU was positively correlated with WI (r = 0.911) and WC (r = 0.910; *p* < 0.05). Notably, WFP/AU did not correlate significantly with WCE.

**Figure 5:**
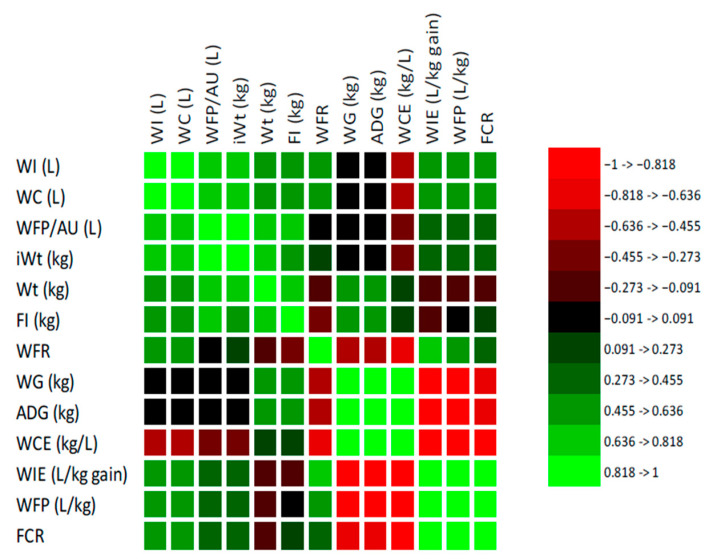
Pearson correlations between water metrics and performance traits in medium-frame beef cattle under intensive production systems.

The Pearson correlation analysis (Figure 5) revealed multiple significant relationships among water and performance indicators in medium-frame beef cattle raised under intensive conditions. There was a perfect positive correlation between WI and WC (r = 1.000; *p* < 0.05). Water intake (WI) was also positively correlated with WFP/AU (r = 0.791), final Wt. (r = 0.558), and FI (r = 0.555), all significant (*p* < 0.05). Notably, WC shared similar significant correlations with WFP/AU (r = 0.799) and FI (r = 0.572). Water footprint per animal unit (WFP/AU) was strongly positively correlated with final Wt. (r = 0.748) and FI (r = 0.755). However, WFP/AU had only a poor positive correlation with growth traits such as WG and ADG (r = 0.089 each), both non-significant (*p* > 0.05). Growth traits showed a strong negative correlation with the FCR, including WG and ADG (r = −0.795; *p* < 0.05). Additionally, WG and ADG were strongly positively correlated with WCE (r = 0.876; *p* < 0.05). Conversely, WG and ADG exhibited strong negative correlations with WIE (r = −0.851) and WFP/kg (r = −0.867), both statistically significant (*p* < 0.05). Water consumption efficiency (WCE) was negatively correlated with WI (r = −0.552) and WC (r = −0.542) and positively correlated with WG and ADG (r = 0.876). In contrast, WIE and WFP had strong positive correlations with FCR (r = 0.900 and 0.942, respectively). The water-to-feed ratio (WFR) had a moderate positive correlation with WI and WC (r = 0.505 and 0.488) and significant positive associations with WIE (r = 0.734) and WFP (r = 0.558) but was negatively correlated with WG and ADG (r = −0.593).

**Figure 6:**
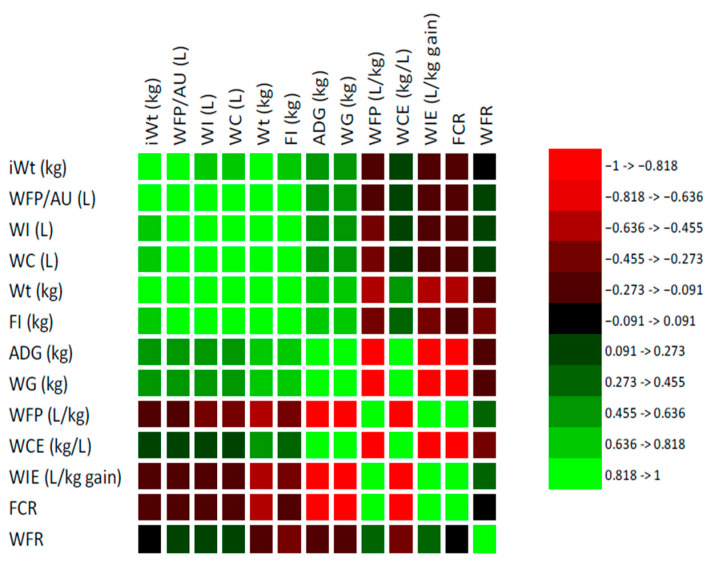
Pearson correlations between water metrics and performance traits in large-frame beef cattle under intensive production systems.

The Pearson correlation coefficients presented in Figure 6 demonstrate key Pearson correlations between water metrics and performance traits in large-frame beef cattle under intensive production systems. Strong and significant positive correlations were found between WI and WC (r = 1.000; *p* < 0.05), WFP/AU (r = 0.992), final Wt. (r = 0.835), and FI (r = 0.845). Water consumption (WC) showed high positive correlations with final Wt. (r = 0.838), FI (r = 0.851), and WFP/AU (r = 0.993). Weight gain (WG) and ADG were positively correlated with WI (r = 0.616), WC (r = 0.620), final Wt. (r = 0.792), and FI (r = 0.697), all statistically significant (*p* < 0.05). Water consumption efficiency (WCE) displayed a strong positive correlation with WG (r = 0.888; *p* < 0.05) and ADG (r = 0.888; *p* < 0.05) and a strong negative correlation with WFP/kg (r = −0.992) and WIE (r = −0.999). Conversely, WIE and WFP showed strong negative correlations with WG (r = −0.884; *p* < 0.05) and ADG (r = −0.929; *p* < 0.05) and strong positive correlations with FCR (r = 0.946; *p* < 0.05) for WIE and (r = 0.956; *p* < 0.05) for WFP. The feed conversion ratio (FCR) was also negatively associated with body weight (r = −0.503 for Wt.), FI (r = −0.271), and WCE (r = −0.945; *p* < 0.05). The WFR showed weak but positive correlations with WI (r = 0.189) and WFP (r = 0.301), but these were not statistically significant (*p* > 0.05).

## 4. Discussion

The evaluation of growth performance and water-use efficiency (WUE) among small, medium, and large-frame beef cattle under intensive production systems revealed significant growth responses and efficiency patterns. The observed order of initial and slaughter body weights, highest in large-frame beef cattle, followed by medium- and then small-frame cattle, reflects the inherent genetic growth potential and mature body size characteristic of each breed. Large-frame beef cattle naturally possess bigger body mass, primarily due to their larger skeletal structure and enhanced muscle development capacity [10,21]. However, the high total weight gain observed in the medium-frame beef cattle, despite their lower mature weight, suggests a more efficient utilization of nutrients during the post-weaning feeding phase. This may be attributed to their favourable growth-to-maintenance ratio, where a greater portion of consumed energy supports gain rather than maintenance, as demonstrated by their high ADG. In contrast, large-frame beef cattle divert a substantial amount of their intake to sustain basic physiological functions, limiting net growth efficiency despite their size [22,23].

The findings from this study indicated that ADG and FCR did not differ significantly between small- and large-frame cattle, further emphasising that despite their higher body weights, large-frame beef cattle are not always the most efficient at converting feed into weight gain. This aligns with findings from Terry et al. [16] and McAllister et al. [20], who underscored that larger-frame beef cattle require higher feed inputs to sustain their growth. The tendency of large-frame beef cattle to reach physiological maturity at heavier weights poses major challenges, such as extended finishing periods, which ultimately increase production inputs. This observation is consistent with the findings of Nyamushamba et al. [24] and Ismail and Al-Ansari [14], who noted that in low-input or extensive production systems, small-frame beef cattle are often preferred due to their lower maintenance feed requirements and quicker attainment of market weight, enhancing overall cost-effectiveness.

However, this is in contrast with the study by Ziegler et al. [25], who indicated that larger-frame beef cattle tend to have higher growth rates compared to smaller-frame beef cattle. This difference in growth patterns necessitates strategic management decisions based on production goals and market demands. Moreover, larger-frame beef cattle require more feed resources, which can impact the carrying capacity of a given environment [25,26,27]. In resource-limited settings, smaller-frame beef cattle may offer advantages due to their lower maintenance requirements and higher relative efficiency [13,14,22]. Therefore, aligning frame size with environmental conditions and resource availability is essential for sustainable production.

Feed intake predictably increased with frame size, as larger animals require more nutrients to maintain homeostasis [21]. Yet, the lack of a significant difference in FI between medium and large frame cattle despite differing weight gains suggests that large-frame beef cattle experienced a higher feed conversion ratio (FCR) [6]. Large-frame beef cattle typically grow at a steady rate but require a longer time to reach market weight, leading to extended finishing periods [25]. Medium-frame beef cattle exhibit faster growth rates and improved feed efficiency, making them an optimal choice for producers seeking a balance between growth and input resources. Small-frame beef cattle reach maturity faster but have lower absolute growth potential and final body weights, which may limit carcass yield.

Water use followed similar trends to FI and Wt., increasing with frame size. This pattern is consistent with the thermoregulatory and digestive demands of larger animals, which require more water for cooling, enzymatic reactions, and metabolic waste excretion [10,15,21,28]. In larger-framed cattle, this demand is heightened due to their higher FI. Inadequate WI can lead to digestive disturbances, reduced FI, and suboptimal WG, all of which negatively influence production performance in beef cattle. These effects are particularly pronounced in large-frame beef cattle, whose higher maintenance requirements are unlikely to be met when WI is limited, thereby further suppressing FI and compromising growth efficiency [24]. This study further observed that the proportional increase in WI and WC compared to WG was not linear, leading to reduced water-use efficiency in large-frame beef cattle. The relative efficiency of medium-frame beef cattle in converting water into gain suggests that their lower maintenance water requirement, coupled with moderate intake, yields a more sustainable water-use profile.

Small- and medium-frame beef cattle used water more efficiently than the large-frame beef cattle, as indicated by their improved WIE and WCE matrices. This is likely due to their optimal rumen function and ability to extract nutrients and water more effectively [16,29]. Their digestive efficiency may enable better hydration and nutrient absorption per unit of feed and water consumed. This aligns with findings by Ahlberg et al. [10] and Dressler et al. [30], who reported that WIE is influenced by both genetic and environmental factors. For instance, cattle with higher WIE tend to perform better in terms of growth, as they utilize water more effectively to convert feed into body mass [16,31]. Optimizing water usage can help minimize the environmental impact of beef production while maintaining healthy growth rates.

While the WFP/kg did not differ significantly among frame sizes, the WFP/AU was notably higher in the large-frame beef cattle (4407 L) compared to the medium-frame (4185 L) and small-frame animals (3822 L). This distinction is important because it highlights that although large animals may be similarly efficient in converting water into weight gain, they accumulate a greater total water use over the production cycle due to their larger body size [3]. Therefore, WFP/AU provides a more holistic indicator of the absolute water resource burden associated with different frame sizes and should be considered when evaluating the sustainability of intensive beef production systems.

The higher WFR observed in large-frame beef cattle can be attributed to their elevated dry matter intake (DMI), which increases their overall water requirements. In this study, large-frame beef cattle recorded an average WFR of 3.33 L/kg feed intake (FI), compared to 3.02 L/kg in the medium-frame and 3.09 L/kg in the small-frame beef cattle. Although the differences are insignificant, the slightly elevated WFR in the large-frame cattle indicates a less efficient water utilization per unit of feed consumed. This trend is consistent with previous research by Golher et al. [28], who reported that larger cattle tend to have higher water requirements relative to their FI due to increased maintenance requirements and thermoregulatory demands. From a sustainability standpoint, maintaining WFR values closer to or below 3.0 L/kg FI has been suggested as a practical benchmark for efficient water use in intensive beef production systems [12,32]. Exceeding this threshold may contribute to higher total water footprint and stress on local freshwater resources, particularly in water-scarce regions. Therefore, optimizing the WFR, especially in large-frame animals, may serve as a valuable strategy to enhance both environmental sustainability and resource efficiency in beef production systems.

The correlation analysis underscored the inverse relationship between resource input and efficiency metrics. As FI and WI increased, WIE and WCE declined, especially in large-frame beef cattle. This confirms the principle that higher absolute consumption does not essentially translate to better productivity and, in fact, may signal inefficiency in energy and water conversion pathways [33]. The medium-frame beef cattle again demonstrated optimal productivity, combining relatively high intake with efficient conversion, thereby enhancing sustainability. A remarkably negative correlation between FI and WI was observed in the small-frame beef cattle during the grower phase. This atypical relationship may be due to physiological factors such as greater digestive efficiency or altered ruminal kinetics, allowing small-frame cattle to extract sufficient nutrients from feed without requiring proportional increases in WI [16,34]. Additionally, their lower thermoregulatory demand and maintenance requirements during this stage may independently reduce water requirements. Behaviourally, it is also plausible that small-frame cattle shift their intake priorities based on environmental conditions or stabilizing growth pathways, leading to reduced voluntary WI despite increased FI [35]. Further research is necessary to confirm and expand on these potential mechanisms.

Despite this study’s insights, certain limitations should be acknowledged. First, although animals were stratified by frame size, breed effects may confound the results. Future studies that explicitly control for breed are recommended. Second, this study was conducted in a single intensive production system under uniform environmental conditions, which may limit generalization to extensive or pasture-based systems. Addressing these limitations in future research will be essential to enhance the robustness and applicability of the findings.

## 5. Conclusions

This study highlights the significance of water footprint in intensive beef production systems and its impact on growth performance and environmental sustainability. The findings demonstrate that large-frame beef cattle exhibit higher absolute growth and water intake but impose greater environmental costs due to increased resource demands. In contrast, small-frame cattle show superior water-use efficiency, while medium-frame cattle present an optimal balance between growth performance and resource efficiency. The strong correlations between feed intake (FI), water intake (WI), and weight gain (WG) emphasize the interdependence of nutrition and hydration in cattle performance. Water intake efficiency (WIE) and water consumption efficiency (WCE) were found to be critical indicators for assessing cattle efficiency under intensive production, with smaller-frame and medium-frame cattle exhibiting superior resource conversion rates. Additionally, water footprint per animal unit (WFP/AU) highlights the higher environmental impact of larger-frame cattle, reinforcing the need for sustainable management practices. These findings suggest that optimizing frame size selection, improving feed efficiency, and implementing strategic water management practices can enhance the sustainability of beef production systems in water-scarce regions. Future research should explore genetic and management interventions to further improve WUE while maintaining high growth performance. By aligning production strategies with environmental sustainability goals, the beef industry can contribute to more resilient and resource-efficient livestock systems.

## Figures and Tables

**Table 1:** Feed ingredient composition per 1000 kg total mixed ration (as-fed basis).

Feed Ingredient (kg)	Starter	Grower	Finisher
Hominy chop	630	670	690
Eragrostis hay	200	180	160
Soya oilcake	80	60	60
Molasses	60	60	60
Limestone	15.0	15.0	15.0
Urea	8.0	9.0	9.0
Salt	5.0	5.0	5.0
Vit/mineral premix	1.9	1.8	1.6
Estimated nutrient specifications (%)			
DM	92.35	93.81	93.13
TDN	74.22	74.69	75.26
NE (MJ/kg)	6.81	6.85	6.91
CF	8.41	7.69	7.08
CP	13.72	13.39	13.51
Ca	6.98	6.86	6.79
P	3.13	3.10	3.14

**Table 2:** Means and LSDs of overall growth performance of beef cattle under intensive production systems.

Frame Size			
Measurements	Small	Medium	Large
Growth performance			
Wt_i_. (kg)	159.77 ^c^ ± 23.65	228.41 ^b^ ± 23.65	265.14 ^a^ ± 23.65
Wt_f_. (kg)	292.14 ^c^ ± 27.27	383.46 ^b^ ± 27.27	412.73 ^a^ ±27.27
FI (kg)	813.68 ^b^ ± 47.03	1025.21 ^a^ ± 47.03	1021.59 ^a^ ± 47.03
WI (L)	2510.64 ^c^ ± 156.3	3095.64 ^b^ ± 156.3	3394.09 ^a^ ± 156.3
WC (L)	2572.88 ^c^ ± 158.8	3174.07 ^b^ ± 158.8	3471.88 ^a^ ± 158.8
WG (kg)	132.36 ^b^ ± 15.11	155.05 ^a^ ± 15.11	142.59 ^a^ ± 15.11
Efficiency measures			
ADG (kg/day)	1.26 ^b^ ± 0.144	1.48 ^a^ ± 0.144	1.41 ^ab^ ± 0.144
FCR (kg feed/kg gain)	6.30 ^a^ ± 0.679	6.66 ^a^ ± 0.679	6.97 ^a^ ± 0.679
WFR (L/kg FI)	3.09 ^b^ ± 0.130	3.02 ^b^ ± 0.130	3.33 ^a^ ± 0.130
WIE (L/kg gain)	19.37 ^b^ ± 2.179	20.15 ^b^ ± 2.179	23.15 ^a^ ± 2.179
WCE (kg gain/L)	0.051 ^a^ ± 0.005	0.049 ^a^ ± 0.005	0.042 ^b^ ± 0.005
WFP/kg (L/kg gain)	29.51 ^a^ ± 3.074	27.21 ^a^ ± 3.074	30.01 ^a^ ±3.074
WFP/AU (L)	3822 ^c^ ± 197.22	4185 ^b^ ± 197.22	4407 ^a^ ± 197.22

^a,b,c^ Rows with different superscripts are significantly different at *p* < 0.05. Wt_i_: initial weight, Wt_f_.: animal final weight, FI: feed intake, WI: water intake, WC: water consumption, WG: weight gain, ADG: average daily gain, FCR: feed conversion ratio, WFR: water to feed ratio, WIE: gain to water efficiency, WCE: water to gain efficiency, WFP/KG: water footprint per kg gain, WFP/AU: water footprint per animal unit.

## Data Availability

The Tshwane University of Technology (TUT) remains the owner of any intellectual property resulting from this study. No information is allowed to be used without the prior permission of TUT.
